# Determinants of Pair-Living in Red-Tailed Sportive Lemurs (*Lepilemur ruficaudatus*)

**DOI:** 10.1111/j.1439-0310.2012.02033.x

**Published:** 2012-05

**Authors:** Roland Hilgartner, Claudia Fichtel, Peter M Kappeler, Dietmar Zinner

**Affiliations:** * Behavioral Ecology and Sociobiology Unit, German Primate CenterGöttingen, Germany; † Department of Experimental Ecology, University of UlmUlm, Germany; ‡ Cognitive Ethology Laboratory, German Primate CenterGöttingen, Germany

## Abstract

Pair-living and a monogamous mating strategy are rare and theoretically unexpected among mammals. Nevertheless, about 10% of primate species exhibit such a social system, which is difficult to explain in the absence of paternal care. In this study, we investigated the two major hypotheses proposed to explain the evolution of monogamy in mammals, the female defence hypothesis (FDH) and the resource defence hypothesis (RDH), in red-tailed sportive lemurs (*Lepilemur ruficaudatus*), a nocturnal primate from Madagascar. We analysed behavioural data from eight male–female pairs collected during a 24-mo field study to illuminate the determinants of pair-living in this species. Male and female *L. ruficaudatus* were found to live in dispersed pairs, which are characterised by low cohesion and low encounter rates within a common home range. Social interactions between pair partners were mainly agonistic and characterised by a complete absence of affiliative interactions – body contact was only observed during mating. During the short annual mating season, males exhibited elevated levels of aggression towards mates, as well as extensive mate guarding and increased locomotor activity. In addition, males were exclusively responsible for the maintenance of proximity between pair partners during this period, and they defended their territories against neighbouring males but not against females. Together, these results point towards the importance of female defence in explaining pair-living in *L. ruficaudatus*. We discuss the spatial and temporal distribution of receptive females in relation to the female defence strategies of males and suggest possible costs that prevent male red-tailed sportive lemurs from defending more than one female.

## Introduction

Most mammals have a mating system characterised by either polygyny (successful males mating with several females and females with only one male) or polygynandry (members of both sexes mating with multiple partners) (Wittenberger & Tilson 1980; Clutton-Brock 1989). In <5% of mammal species, however, individuals mate with only one partner over one or several reproductive cycles (Kleimann 1977; Clutton-Brock 1989; Dobson et al. 2010). Their mating system can be referred to as monogamy and typically corresponds to a social organisation where a male and a female form a stable social unit, that is, that they are pair-living (social or behavioural monogamy) (Kappeler & van Schaik 2002; van Schaik & Kappeler 2003; Schubert et al. 2009).

Given the physiological constraints of internal gestation and lactation, mammalian mating strategies evolved under a particularly strong asymmetry between the sexes (Williams 1966). Female mammals generally have lower potential reproductive rates and make a much higher parental investment than males. Male mammals can maximise their reproductive success by mating polygynously, so that it is not surprising that the majority of mammal species evolved a polygynous mating system (Trivers 1972; Clutton-Brock 1991; Cohas & Allainé 2009).

Whenever biparental care is obligate or paternal care improves male reproductive success, as is the case in most bird species (Birkhead & Møller 1996; Møller 2003), pair-living and monogamy can be explained adaptively also from the male perspective (Trivers 1972; Mock & Fujioka 1990; Gubernick 1994; Iwasa & Harada 1998; Møller & Cuervo 2000; Schülke 2005; Borries et al. 2010). Various forms of such paternal care behaviours have been cited to explain monogamy in a few mammals (e.g. owl monkeys *Aotus* spp*.:*Wright 1985; Fernandez-Duque 2011; Callitrichidae: Dunbar 1995; California mouse *Peromyscus californicus*: Cantoni & Brown 1997; fat-tailed dwarf lemur *Cheirogaleus medius*: Fietz 1999; rock ringtail possum *Petropseudes dahli*: Runcie 2000), which represent, however, apparently only a minority of species (van Schaik & Dunbar 1990; Komers 1996; Komers & Brotherton 1997; Fuentes 2002). In addition, paternal care in mammals can exist in the absence of monogamy (Wright 1990; Buchan et al. 2003), and females may also choose particular males because of the benefits they provide to either themselves or their offspring in the form of protection from predation, infanticide or harassment (Gowaty 1996; Borries et al. 2010).

Several hypotheses have been proposed to explain the evolution and/or maintenance of monogamy in mammals (see Orians 1969; Kleiman 1977; Wittenberger & Tilson 1980; Reichard & Boesch 2003; Borries et al. 2010; Dobson et al. 2010). The two main hypotheses are the female defence hypothesis (FDH) and the resource defence hypothesis (RDH). The FDH assumes that dispersal of females is determined by the temporal and spatial distribution of resources and that males map themselves onto the distribution of females, defending or monopolising as many females and/or female home ranges as possible (van Schaik & van Hooff 1983; Altmann 1990; Komers & Brotherton 1997; Palombit 1999; Norscia & Borgognini-Tarli 2008; Wolovich et al. 2010). Female defence is the optimal male strategy if females are so widely distributed in space or exhibit such highly synchronised oestruses that economic defence of more than one female at a time is not feasible (Emlen & Oring 1977; Nunn 1999; Dunbar 2000; Dobson et al. 2010). Males adopting a roving strategy would not achieve higher reproductive success than males focusing on only one mate (van Schaik & Dunbar 1990; Dunbar 2000; Schubert et al. 2009).

Under the RDH, males monopolise resources important to females by defending a territory instead of defending females directly (Emlen & Oring 1977; Wrangham 1979; van Schaik & Dunbar 1990). Hence, male reproductive success is limited by the females’ choice of resource access. In fact, a phylogenetic analysis revealed that mammalian monogamy evolved where females were solitary and occupied small, exclusive ranges, enabling males to monopolise them (Komers & Brotherton 1997; but see Shultz et al. 2011). If males pursue resource defence as a mating strategy, pairs should emerge whenever males are unable to maintain territories that can support more than one female. Resource defence should be especially likely in species where females are subject to high energetic demands during gestation and lactation (Brockelman & Srikosamatara 1984). Territorial defence by males decreases food competition among females and makes female reproductive success dependent on male resource holding potential (Parker 1974). However, high-quality territories should attract and support multiple females, even if intrasexual aggression between females is high (Orians 1969; Davies 1989). These two hypotheses are not mutually exclusive and a mixture of several causes and functions of pair-living is possible (Fuentes 2002; Reichard & Boesch 2003; Dobson et al. 2010).

Phylogenetic reconstructions revealed that pair-living in primates evolved several times independently in all major radiations – most likely from ancestors with a promiscuous mating system (van Schaik & Kappeler 2003; Muhlberger 2011; Shultz et al. 2011). Among pair-living primates, there seems to exist considerable variation in the degree of spatial cohesiveness between pair partners (Müller & Thalmann 2000; van Schaik & Kappeler 2003; see also Cohas & Allainé 2009). Hence, species are classified as dispersed pairs when pair partners share a home range but are not consistently associated during their period of activity (e.g. *Phaner furcifer:*Schülke & Kappeler 2003; *Lepilemur edwardsi:*Rasoloharijaona et al. 2006). In contrast, species are considered as living in cohesive pairs whenever pair partners are permanently spatially associated, travel cohesively and interact frequently (e.g. *Hylobates lar*: Reichard 1995a; Asensio et al. 2011).

The red-tailed sportive lemur, *Lepilemur ruficaudatus*, is a small (780 g), nocturnal folivorous strepsirrhine primate restricted to the dry deciduous forests of central western Madagascar. Pairs maintain stable territories of around 1 ha for several years (Ganzhorn & Kappeler 1996; Zinner et al. 2003). Their mating season is limited to only a few weeks in late May and June (Hilgartner et al. 2008). Extra-pair copulations may occur at very low rates (unpublished data). Single infants are born at the beginning of the rainy season in late November and are weaned about 2 mo later (Hilgartner et al. 2008). Males do not exhibit any direct paternal care (Hilgartner et al. 2008). Although the mating season of these sportive lemurs is very short, pairs share a common home range year-round.

To explore possible causes for pair-living in red-tailed sportive lemurs, we deduced specific predictions about the spatial and social relationships among pair partners and neighbouring individuals from RDH and FDH (summarised in Table 1) and test them with spatial and behavioural data collected from eight sportive lemur pairs over a 24-mo period.

**Table 1:** List of predictions derived from female defence (FDH) and resource defence (RDH) hypotheses

Female defence (FDH)	Resource defence (RDH)	Test
Proximity between pair partners mainly during pre-mating and mating season	Proximity between pair partners does not differ among reproductive seasons	Comparison of cohesiveness and interindividual distances among different reproductive seasons
Mainly males responsible for within-pair proximity	Neither males nor females are responsible for within-pair proximity	Hinde index for proximity
Males are aggressive against strange males not against strange females	Males are aggressive against strange males and females	Analysis of observed encounters with neighbours
Home range use and travel distance differ between mating and non-mating season	Home range use and travel distance do not differ between mating and non-mating season	Comparison of home range use and travel distances during mating and non-mating season
Males are not able to defend more than one home range	No specific prediction	Calculation of defendability indices D (Mitani & Rodman 1979) and M (Lowen & Dunbar 1994) and analysis of oestrous synchrony

## Methods

### Study Site

This study was carried out in Kirindy Forest, western Madagascar (44°39′E, 20°03′S), where the German Primate Center (DPZ) operates a field research station (Kappeler & Fichtel 2012). The local climate is characterised by pronounced seasonality with a short rainy season from December to March, followed by a longer dry season with little or no rain from April to November (Sorg et al. 2003). The forest is dense, and most tree species do not exceed 20 m in height (Ganzhorn & Sorg 1996).

The study area (locally known as N5) is located within a 12 500 ha forest concession of the Centre National de Formation, d’Etudes et de Recherche en Environnement et Foresterie (CNFEREF) Morondava. The study area was defined by the boundaries of a systematic grid system. Within a 500 × 500 m core area, small trails were established every 25 m in both north-south and east-west directions, surrounded by additional trails at 50 and 100 m intervals along three edges of the core area. Along its western border, former logging trails (200 m long at 100 m intervals) were used for radio-tracking whenever necessary. Each trail intersection is marked with a plastic tag for orientation. The entire grid system was mapped, and coordinates of each intersection were determined.

### Capture and Marking

Between 1995 and 2004, a total of 87 individuals were captured from their sleeping sites in hollow trees during the day. Potential sleeping trees were initially located by transect walks, and animals were caught by hand or by placing a live-trap at the tree hole entrance. Animals were briefly anaesthetised with GM2 (Rensing 1999) and marked with a unique subcutaneously injected transponder (Trovan, Usling, Germany). Adult animals captured within the core area of our study site were equipped with 9 g radio collars (Biotrack, Wareham Dorset, UK), which is <3% of the animal’s body mass. Radio collars with unique frequencies were fitted around the neck. All radio collars were removed after the end of the study. Infants and subadults were marked with unique visual cues by shaving parts of their tail. Adult males and females forming 8 pairs were fitted with radio collars between 2002 and 2004.

### Ethical Statement

None of the trapped or radio-collared animals showed any sign of discomfort or were restricted in their mobility or other behaviour. The study and the applied methods have been approved by the Commission Tripartite CAFF (Madagascar).

### Data Collection

Data were collected on eight pairs that were observed continuously for 24 mo between 2002 and 2004, totalling >2000 observation hours. Each pair was observed for at least one reproductive cycle, including pre-mating (February–April), mating (May–June), gestation (June–October) and birth/weaning (November–January) (Hilgartner et al. 2008). We followed radio-tagged animals with radio-tracking equipment from Telonics (Mesa, AZ, USA). We limited observations mainly to the first 8 h of the night (1800–0200 h), because in a pilot study, we did not find a difference between the first and the second part of the night with respect to activity budget and travel distances. Within these 8 h, we collected data on three pairs (each pair was observed for 2 h). The 2-h blocks were systematically rotated among the observed pairs to control for differences between observation time and nights. We observed the animals with the aid of a headlamp and occasional use of a strong flashlight and binoculars. We attempted to observe all 16 adult individuals for equal periods of time. Together with a Malagasy field assistant, R.H. followed both pair partners simultaneously for 2 h, using focal animal sampling (Altmann 1974). At 5-minute intervals, the exact location, as well as the behavioural state (feeding; resting; locomotion) of each focal animal was recorded (instantaneous sampling, Altmann 1974). Observer distance from the focal animals was between 1 and 15 m. We recorded whether animals were out of sight at the time of instantaneous sampling of behaviour. Analyses and calculation of feeding time was based on the number of intervals animals were in sight. Social interactions between pair partners and among neighbours were recorded by all occurrences. Additionally, sleeping sites of *L. ruficaudatus* were marked, and members of sleeping associations were identified during the day by detecting their radio or transponder signal.

### Data Analyses

Analyses of spatial data were performed with the Animal Movement extension for ArcView^©^ (Hooge & Eichenlaub 1997). We used both kernel home ranges (KHR; Worton 1989) and minimum convex polygons (MCP) to describe the overall home range size and to calculate home range overlap. Home range overlap was calculated for both, pair partners and same-sexed neighbours. We used the MCP method, which tends to overestimate home range size to enable comparisons with published data for other species. Our spatial analyses are based on 873–1452 data points or fixes for each of 16 individuals. For a detailed description of the calculation of home range saturation and centres, see Zinner et al. (2003).

To estimate cohesiveness between pair partners, we calculated the percentage of time pair partners spent in various distance categories, ranging from 0 to 180 m. We used an intrapair distance of <10 m as the criterion for cohesiveness. We chose this distance because it most likely permits visual contact between partners, and pair partners show higher behavioural synchrony in this distance category (Fichtel et al. 2011). Cohesiveness was compared across the annual reproductive cycle, pooling data for the pre-mating and mating seasons (in the following called ‘mating season’), as well as for gestation and birth seasons (in the following called ‘non-mating season’).

Hinde indices were calculated to investigate responsibility for the maintenance of spatial proximity within pairs (Hinde & Atkinson 1970). Values range between 1 and −1, with values between −0.1 and 0.1 indicating equal responsibility for the maintenance of spatial proximity.

We compared observed encounter rates of pair partners with expected encounter rates calculated with a random gas model (Waser 1976):

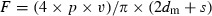


Generally, the expected encounter rate (*F*) depends on the population density (*p*; individuals/area), velocity of the animals (*ν*; m/h), group spread (*s*; maximal distance among group members in metres) and the distance criterion (*d*_m_). In our analysis, we calculated (*p*) for each pair separately as the inverse of the home range, including also exclusively used areas of pair partners (additive home range). Velocity (ν) of animals was the average distance male and female travelled per night, that is, within 10 h. From our 2-h observation protocols, we were able to calculate the average travelled distance per hour. For the distance travelled per night (10 h), these values were multiplied by 10. We defined encounters (distance criterion *d*) as situations in which pair partners approached to within 10 m. We calculated encounter rates separately for each reproductive season and compared them with observed encounter rates in the respective other seasons.

To compare observed encounter rates between neighbouring males with expected encounter rates, we modified the original gas model:

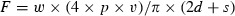


We calculated population density (*p*) for each male–male dyad separately as the inverse of the overlapping home range area. Velocity (ν) of animals was the average distance both males travelled per night. We used the same distance criterion as for pairs. Because both males ranged also in their exclusive areas, we corrected the model for the probability (*w*) that both males were within the overlapping area at the same time. We calculated encounter rates (per half night; 6 h) separately for each reproductive season and compared them with observed encounter rates.

We classified social encounters between individuals into three categories: agonistic, neutral and affiliative. Affiliative behaviour included huddling and grooming. Agonistic behaviour was either aggressive (chase, charge, bite and grab) or submissive (flee, be displaced or jump away) *sensu*Pereira & Kappeler (1997). To determine dominance relationship between pair partners, we only used decided conflicts where one partner showed only submissive behaviour and no aggression and the opponent no submissive behaviour, but aggression. Here, we also consider mate guarding or aggressive coercion of males towards their mates as an indirect form of female defence because females are not able to choose their mates freely (Brotherton & Manser 1997; Palombit 1999; Schülke 2005).

To examine changes of travel distances between mating and non-mating season, we controlled for potentially confounding ecological factors, such as rainfall and temperature and availability of young or adult leaves and abiotic factors. Travel distances in the mating season were compared with travel distances during 3 wk (June) following the mating season. All ecological factors remain fairly constant within these two time periods (Sorg & Rohner 1996; Ganzhorn 2002).

To investigate whether males and females spent more time in the periphery of their home range during the mating season, we also determined the average time focal animals spent in certain distance categories from the centre of their home range as a measure of space use. If individuals spent more time in the periphery during the mating season, the average distance from their home range centres should be larger than during the non-mating season. To examine home range defendability, we calculated the widely used *D* (Mitani & Rodman 1979) and *M* indices (Lowen & Dunbar 1994). Species with *D* values >0.98 and *M* values > 0.08 are considered to be able to defend territories.

We operationally defined periods of oestrus by two criteria: presence of a swollen vulva and mate guarding (Hilgartner et al. 2008). To estimate oestrous synchrony, we calculated days of overlap of oestrus (as defined above) for all female dyads. To test whether neighbouring females were more synchronous than females with more distant home ranges, we correlated distances among females’ home range centres and days of oestrous overlap among females. Statistical analyses were performed with Statistica 9.0 STATSOFT Inc.

## Results

### Home Range Size and Overlap

Average male home range size was significantly larger than that of the corresponding female pair partner (MCP: males 15946 ± 6373 m^2^; females 11773 ± 3095 m^2^; *t* = 3.1; p < 0.05; n = 8; 95% KHR: males 9912 ± 5962 m^2^; females 6581 ± 3773 m^2^; paired *t*-test: *t* = 2.9; p < 0.05; n = 8). Average maximum 95% KHR diameter was 175 ± 31 m.

**Figure 1:**
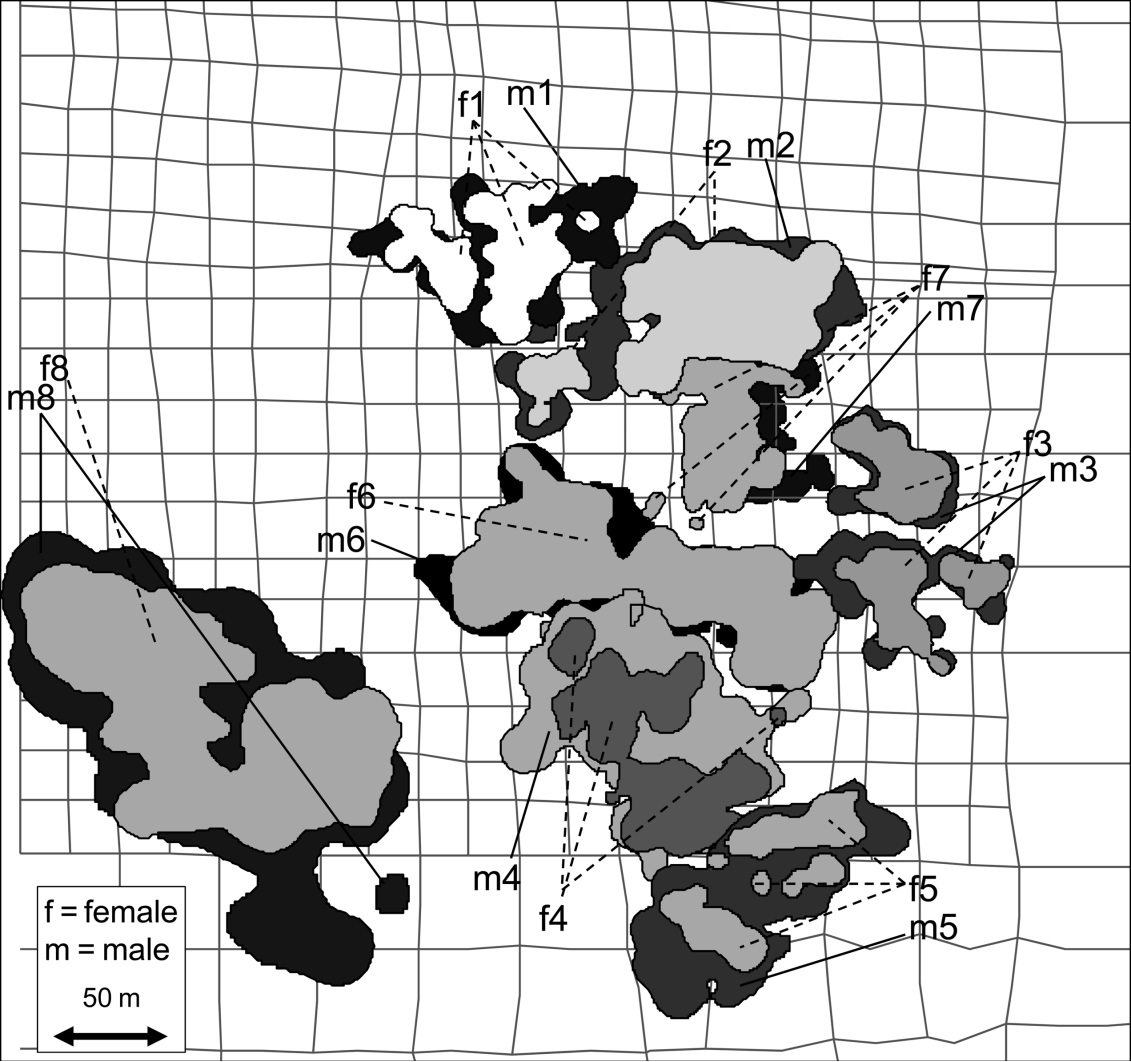
Home ranges of eight *Lepilemur ruficaudatus* pairs in 2002–2004 plotted on a sketch of the grid system of the study area. Shown are Kernel 95% probability home ranges (KHR). Home ranges of females are located within home ranges of the respective male pair partners.

As MCP home ranges were larger than KHRs home ranges, overlap based on MCP home ranges was bigger than the respective overlap based on KHR. Average overlap of 95% KHRs between pair partners was 61.3 ± 13.6% from the male’s perspective and 89.4 ± 8.3% from the female’s perspective (Fig. 1). Overlap between pair partners based on MCPs was 67.6 ± 10.6% (males’ perspective) and 89.0 ± 9.5% (females’ perspective), respectively. Differences between male and female perspectives are because of larger male home ranges. MCP home range overlap between female neighbours was observed for 5 dyads with an average overlap of 16.9 ± 12.6%. For KHRs (95%), we observed only 3 dyads with an average overlap of just 1.8 ± 1.7%. Average home range overlap between neighbouring males was slightly larger than overlap between neighbouring females (MCPs, 6 dyads, 15.7 ± 12.7%; KHRs, 5 dyads, 2.3 ± 1.5%). Home range overlap between neighbouring male–female dyads also occurred (female’s perspective: MCP, 13 dyads, 17.4 ± 10.3%; KHR 95%, 8 dyads, 1.8 ± 1.3%; male’s perspective: MCP, 13.8 ± 8.5%; KHR 95%, 2.0 ± 1.8%).

### Cohesiveness Between Pair Partners

**Figure 2:**
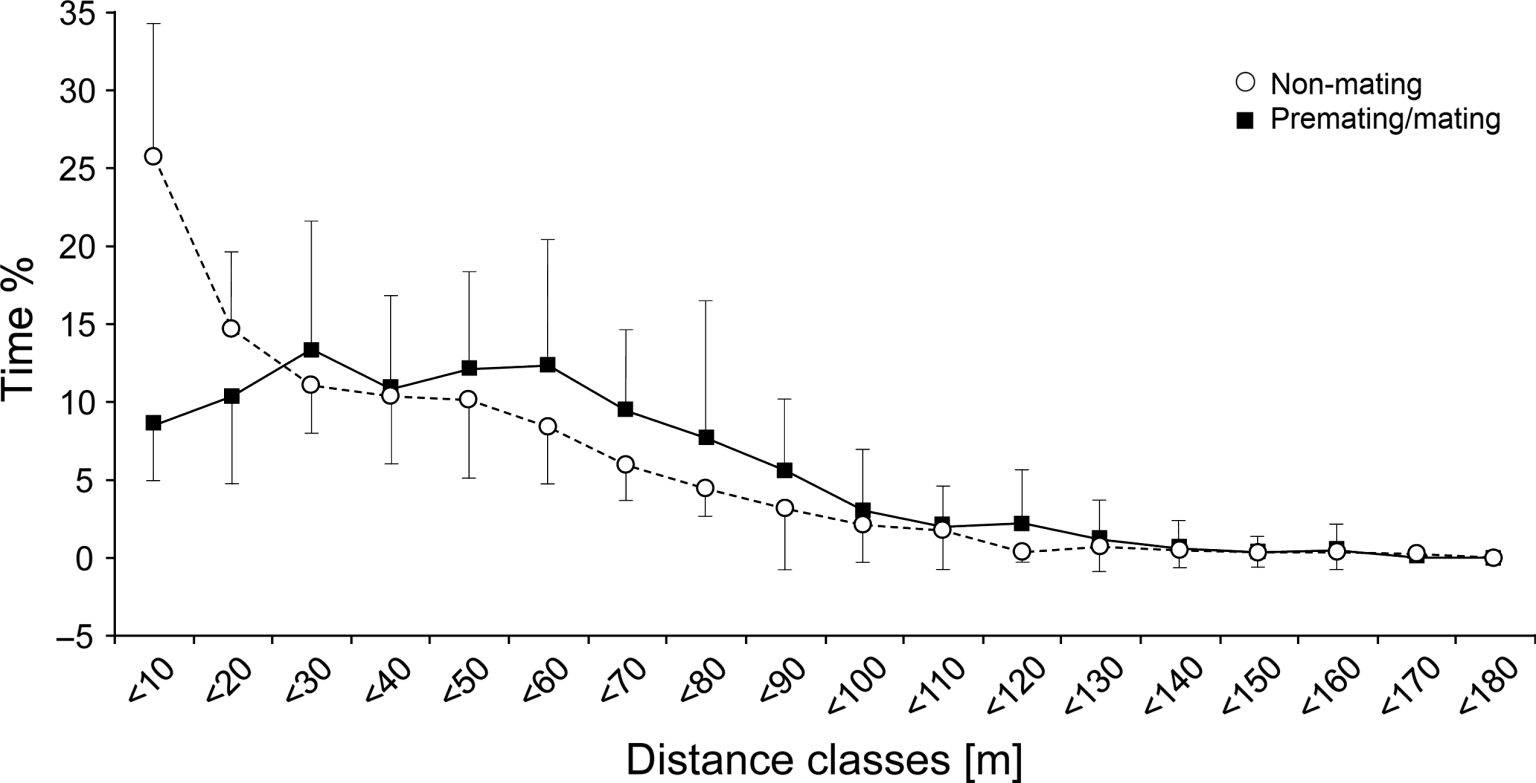
Interindividual distances between pair partners during pre-mating/mating and non-mating season (n = 8 pairs).

Pair partners were found in distances of between 0 and 180 m from each other (mean 43.5 ± 5.9 m). However, the percentage of time pair partners spent in certain distance categories depended on reproductive season (Fig. 2). During the pre-mating and mating seasons, males spent on average 25.7 ± 7.9% of the time at a distance of <10 m from the female, but during the non-mating season, this proportion decreased to only 8.8 ± 2% of the time (*t*-test-dependent samples: *t* = 7.2, p < 0.01, n = 8).

Hinde indices on the basis of approach/leave interactions, irrespective of the behavioural context, indicate that in all eight pairs, males were responsible for the maintenance of proximity throughout the year (Table 2; sign test: p < 0.05).

**Table 2:** Hinde index for proximity calculated from the male’s perspective

Pair	Approach [%]	Leave [%]	N	Hinde	Mop
1	96.3	33.3	27	0.63	Male
2	87.5	43.8	16	0.44	Male
3	85.0	55.0	20	0.30	Male
4	83.3	46.7	30	0.37	Male
5	79.2	20.8	24	0.58	Male
6	92.0	32.0	25	0.60	Male
7	78.6	50.0	28	0.29	Male
8	92.0	28.0	25	0.64	Male
Mean	86.7	38.7	24.4	0.48	

*N* sum of all approach and leave interactions; *mop* responsible for the **m**aintenance **o**f **p**roximity.

### Encounter Rate, Type of Encounter and Dominance Relationship within Pairs

In total, we observed 255 social encounters between pair partners. The average encounter rate was higher during the mating season compared with the non-mating season (3.5 ± 0.6/6 h vs. 1.6 ± 0.7/6 h; *t*-test-dependent samples: *t*= −8.82, p < 0.0001). Furthermore, during the mating season, observed encounter rates were significantly higher than expected by chance (as estimated by the gas model, *t*-test-dependent samples: *t* = −7.73 p < 0.001, n = 8). Observed encounter rates in all other seasons did not deviate from random expectations (*t*-test-dependent samples: *t* = −1.83, p = 1.1092, n = 8).

On average, 47.3 ± 7.4% of the encounters between pair partners involved agonistic behaviour, with 76 of 120 of these conflicts being decided. On average, half (49.7 ± 15.9%) of all agonistic interactions were won by males, but during the mating season, this rate increased to 87.1% (N = 31). In contrast, males lost most of the conflicts (78.9%; N = 38) during the birth season. During the rest of the non-mating season, agonistic encounters between pair partner were rare and wins were equally distributed between pair partners (N = 7 conflicts: winner male 3; winner female 4). No affiliative interactions between pair partners, such as grooming, were observed throughout the study. The only non-agonistic situation in which males and females had body contact was mating.

### Encounter Rates and Type of Encounters among Neighbours

Males encountered neighbours on average once every two nights (every 19.6 h of observation time), but females met neighbours significantly less often (once every five nights or every 52.6 h; *t*-test-dependent samples: *t* = 2.56; p < 0.05; n = 8). We detected no evidence for roaming males during the study period and, hence, no encounters between focal and roaming males were observed.

Home range overlap among seven male neighbour pairs permitted us to calculate expected encounter rates based on area of overlap. The observed encounter rates between neighbouring males did not differ from expected random encounter rates for any season (Wilcoxon matched-pairs signed-ranks tests, n = 7: dry season *Z* = 0.944, p = 0.345; birth season *Z* = 1.352, p = 0.176; mating season *Z* = 1.014, p = 0.310).

In 95% (N = 23) of encounters between neighbouring males, aggression was involved. Encounters between neighbouring females and males involved aggression in only 23% (N = 22). Encounters between neighbouring females were only rarely observed, and in one of three encounters, aggression was observed. Sex of the opponent therefore had a significant effect on the probability of agonistic behaviour (χ^2^= 18.55, df = 1, p < 0.0001).

### Comparison of Travel Distance and Space use Between Pair Partners

Throughout the year, males travelled on average 32.0 ± 12.8% longer distances than their female partners (males: 90.9 ± 15.6 m/h; females: 61.3 ± 12.5 m/h; *t*-test-dependent samples: *t* = 5.84, p = 0.0006; n = 8). Males reduced their travel distances in the 3 wk following the 3-wk mating season on average by 40.2 ± 34.2%. In the same period, females reduced their travelling on average by 21.7 ± 24.4%. The difference between the sexes was not significant (*t*-test-dependent samples: *t* = 1.29, p = 0.2371; n = 8).

**Figure 3:**
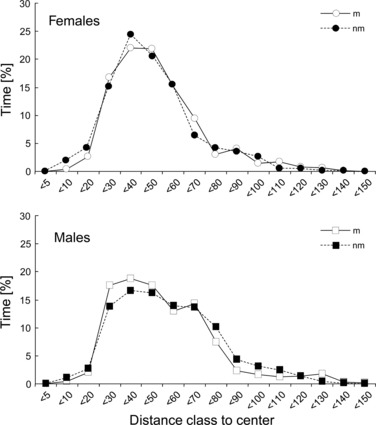
Mean percentage of time females and males spent in certain distance categories to the centre of their respective home range during mating (m) and non-mating (nm) season (females n = 8; males n = 8).

Males and females also did not show differences in their distributions of space use among seasons, that is, they did not spend more time in the periphery of their home ranges in either season (Fig. 3; Kolmogorov–Smirnov test; females: *Z* = 0.354, p = 1.000; males: *Z* = 0.706, p = 0.699).

### Monopolisation Potential and Defendability in Relation to Home Range Size, Travel Distance and Oestrous Synchrony

Both territory defendability indices (*D* = 8.1; M = 0.72) indicate that *Lepilemur ruficaudatus* should be able to defend territories. Average time lag of behavioural oestrus among the eight observed females was 3.4 ± 2.8 d. As we correlate here two distance matrices (spatial distance between activity centres of 8 females and temporal distance between oestrus of the same 8 females), we performed a Mantel test by ranks. We did not find evidence for neighbouring females to experience a more synchronous behavioural oestrus than females living in more distant home ranges (Mantel test by ranks with 40320 permutations (SsS 2.0): *R* = 42 802; *p*_1_ = 0.7541; *p*_2_ = 0.2531).

## Discussion

*Lepilemur ruficaudatus* is pair-living, and a pair occupies a common exclusive home range. Furthermore, our study revealed that the behaviour of males, in particular, changed dramatically during the short annual mating season. At this time, males increased travel distances, stayed in close proximity to females, which is indicative of mate guarding, and showed elevated levels of aggression towards mates, which lead to male dominance during the mating season. In contrast, females were dominant over males during the birth season. Throughout the year, males were responsible for maintaining proximity between pair partners and defended territories mainly against other males but not against females. These results support predictions of the female defence hypothesis.

### Proximate Mechanisms of Pair-Living

Pair partners in *L. ruficaudatus* are rarely in close spatial proximity and rarely interact with each other; a pattern also described for pale fork-marked lemurs, *Phaner pallescens* (Schülke & Kappeler 2003), and a few other mammals (Munshi-South 2007; Cohas & Allainé 2009). Despite these similarities, encounter rates in *P. pallescens* were higher during the non-mating season than expected by the gas model, whereas encounter rates in *L. ruficaudatus* did not deviate from expected values. Assuming that the gas model describes the far end of interindividual spacing within pairs, and encounter rates of *P. furcifer* are interpreted as being rare (Schülke & Kappeler 2003), the even lower encounter rates in *Lepilemur* suggest active avoidance of pair partners.

Avoidance of pair partners can be explained as a consequence of intersexual feeding competition (Schülke & Kappeler 2003). Differences in the degree of avoidance between *P. pallescens* and *L. ruficaudatus* may be a result of their different dietary regimes. *Fork-marked lemurs* are specialised gum feeders that exploit only a small number of tree species (Schülke 2003a). Males and females exploit the same small number of tree individuals within their common home range, which may enhance encounter frequency. Hence, avoidance of pair partners in such a specialised forager may be more difficult than in folivores, such as *L. ruficaudatus* (Pietsch 1998).

Interestingly, social cohesiveness between pair partners appears to vary intensively within the genus *Lepilemur* with frequent affiliate interaction, vocal duetting and common use of sleeping sites in *L. edwardsi* or a solitary lifestyle without vocal communication in *L. mustelinus* (Warren & Crompton 1997; Rasoloharijaona et al. 2003, 2006, 2010; Méndez-Cárdenas & Zimmermann 2009, Fichtel & Hilgartner in press). Vocal and chemical communication may play important roles in mediating individual spacing, but the required data to test their function are not available (Fichtel & Hilgartner in press).

The quality of intersexual encounters is also highly variable among pair-living primates. To our knowledge, *L. ruficaudatus* is the only pair-living primate species for which no form of affiliative interactions among pair partners, such as grooming or huddling, has been reported outside the mating context. In white-handed gibbons, pair partners coordinate their activities, and grooming bouts between males and females make up to 15% of their daily activity (Brockelman & Srikosamatara 1984; Cowlishaw 1992; Reichard 1995b). Attributes of the pair-bond in titi monkeys (*Callicebus* spp.) also include frequent grooming bouts, small interindividual distance and close behavioural coordination (Kinzey 1997; Müller & Anzenberger 2002). In other pair-living lemurs, affiliative interactions have also been frequently observed (Fietz & Dausmann 2003; Schülke 2003b).

Equally striking is the fact that about half of all encounters between pair partners in *L. ruficaudatus* were of an aggressive nature. The quality of non-agonistic interactions resembles that of solitary species with pronounced inter-and intrasexual home range overlap. For *Mirza coquereli,*Kappeler (1997) reported few affiliative interactions among individuals in general and disproportionately many aggressive encounters between adult males and females. A similar pattern was observed in *Microcebus murinus,* where encounters between male and females were mainly aggressive, and grooming was only observed between female dyads or adults and subadults (Eberle & Kappeler 2004).

In primates, it has been long assumed that pair-living as a type of social organisation evolved from a solitary ancestor (Müller & Thalmann 2000; Low 2003), but recent phylogenetic reconstructions suggested that it was derived from group-living (Shultz et al. 2011). The different pattern of cohesiveness, encounter rates and relationship qualities among pair-living species was considered to represent different evolutionary stages in the transition from a solitary to a pair-living lifestyle (van Schaik & Kappeler 2003). Some morphological traits, such as male-biased sexual canine dimorphism and a seasonal increase in testes volume in *L. ruficaudatus* (Zinner et al. 2003), are not predicted for pair-living species and are compatible with a recent transition from either a solitary or group-living ancestor. Alternatively, this set of traits may reflect an adaptation to high opportunities for extra-pair matings (Munshi-South 2007; Cohas & Allainé 2009).

### Why Defend Only One Female?

Socio-ecological theory (Emlen & Oring 1977) suggests that unfavourable distributions of fertile females in either time or space are the main constraints on male monopolisation potential. Comparative analyses of home range size in mammals have indeed revealed that female space use is a fundamental predictor for pair-living (Komers & Brotherton 1997; Dobson et al. 2010; Carnes et al. 2011). Although mating was highly seasonal, we did not find evidence for females synchronising their oestruses. Instead, *Lepilemur* females exhibited a comparatively high degree of home range exclusivity (cf. Fietz 1999; Schülke 2003b; Schubert et al. 2009; Dobson et al. 2010; Wright et al. 2010). In fact, home range overlap among females was virtually absent and neighbouring females rarely met, indicating high levels of female intrasexual avoidance or resource competition. Thus, as in other mammals, a certain degree and combination of home range size, home range overlap and intra-and intersexual aggression may represent a fundamental threshold for *Lepilemur* males in their ability to monopolise or roam over territories of several females (see also Komers & Brotherton 1997; Rathbun & Rathbun 2006; Schubert et al. 2009).

In several other pair-living mammals, high intrasexual aggression (gibbons: Brockelman & Srikosamatara 1984; Mitani 1984; golden lion tamarins: Baker & Dietz 1996) or dispersion of females (elephant shrews: Rathbun 1979; beavers: Sun & Müller-Schwarze 2003) is considered as typical traits that favoured the evolution of pair-living. In Madagascar, resource scarcity may promote pair-living because it enhances female spacing (Wright 1999). To determine whether females’ distribution limits male monopolisation potential, it is important to consider the defendability of territories. Defendability indices for *L. ruficaudatus* did not indicate that females are over-dispersed because males should be able to defend territories of up to eight females. This value is comparable to defendability indices of other pair-living primates, where males could defend areas large enough to include the ranges of 4–7 females (van Schaik & Dunbar 1990; Schülke 2005). Because in some solitary species, males’ ranges cover those of up to 20 females (Kappeler 1997; Eberle & Kappeler 2004), the defendability threshold should be considered with caution, however. The defendability index may already reflect the consequence of mate competition, and they do not include possible additional costs and constraints of territorial defence or roaming, respectively (Promislow 1992).

We therefore propose several additional costs that could prevent males of *L. ruficaudatus* from monopolising more than one female or from adopting a roaming strategy. First, behavioural oestrus of females is short, and mating is probably restricted to only one night per year (Hilgartner et al. 2008). Therefore, information about female reproductive state is crucial for males. We assume that males seek and gain information about the reproductive state of females because encounter rates within pairs increased during pre-mating and mating seasons. Moreover, males were responsible for the maintenance of proximity and showed intense mate guarding during behavioural oestrus, as is also the case in other species (e.g. Schubert et al. 2009). However, obtaining this information seems costly for male sportive lemurs because of high aggression between pair partners. Hence, monopolising more than one female would increase energetic costs of males considerably because of aggression from several females and may lead to less exclusive and precise information about female reproductive status (Ribble 2003).

Second, mate competition in *L. ruficaudatus* seems to be already intense for males defending only one female. Males encountered neighbours every second night, and in about 95% of encounters, aggression was observed. Moreover, we witnessed one extra-pair copulation, suggesting that extra-pair mating options influence the trade-off between mate guarding and roaming. Potential costs of roaming accrued from additional travel and an increased risk of injury may constrain this type of behaviour.

Third, *L. ruficaudatus* is vulnerable to a range of terrestrial and aerial predators (Rasoloarison et al. 1995; Fichtel 2007), both at night and during the day, which they spend in tree holes (Schülke & Ostner 2001; Rasoloharijaona et al. 2008; Fichtel in press). Predation risk should be higher for males that travel more because they are more exposed and spend more time in less familiar areas with reduced knowledge about suitable day shelters. A more risk-averse strategy characterised by reduced roaming that may result from these and other constraints was also proposed to explain pair-living in other mammals (e.g. Kirk’s dik dik: Brotherton & Manser 1997; Brotherton & Komers 2003; elephant shrews: FitzGibbon 1997; Ribble 2003).

### Does Pair-Living Represent a Dilemma for *Lepilemur* Females?

Given that an observed mating system or type of social organisation may represent the outcome of a compromise between male and female strategies, it is of interest to consider the females’ perspective in this context, as well. We assume that the options for female choice in *L. ruficaudatus* are restricted. First, in four cases of re-pairing (following predation of mates), we found no evidence that females tried to repel new immigrant males. Second, location of territories and pair composition remained stable for several years (Zinner et al. 2003); that is, there is no evidence for ‘divorce’ (as in alpine marmots: Lardy et al. 2011). Third, mate guarding of males is intense, and males dominate females during the short mating season. Fourth, extra-pair copulations are rare, and thus, females may have only limited control over which male they live and mate with.

Females may also reap benefits from being paired with a male. They may face reduced sexual harassment by strange males, which has been shown to be costly to oestrous females in promiscuous species (Borries et al. 2010). They are also likely to benefit from reduced feeding competition because additional males are excluded from their home range (Schülke 2005). In addition, serial pair-living, as suggested for owl monkeys, where intruding males are able to expel resident males (Fernandez-Duque 2011), was also observed in *L. ruficaudatus.* Although some females were paired with one male over a period of at least four years, others (n = 4) lived with two males successively within four years. In all observed cases, death of the previous pair partner was responsible for the appearance of a new male. Hence, serial pair-living could at least compensate for a reduced genetic variability of offspring, if not for a reduction in opportunities for female choice.

## Conclusions

In summary, this study underlines the value of the female defence hypothesis when investigating the evolution of pair-living in mammals. Ranging patterns of females as well as a short mating season affect monopolisation potential of males. However, these factors *per se* cannot fully explain the evolution of pair-living in *L. ruficaudatus*. In addition to emphasising these constraints, we suggest that increased inter-and intrasexual aggression as well as a higher predation risk and energetic constraints may prevent males from adopting a roaming strategy. Minimisation of risks, and hence a minimisation of the variance in mating success, may explain why most males focus their reproductive effort on only one female in *L. ruficaudatus*. Our study also highlights the fact that lifetime reproductive success, as well as the relative importance of benefits and costs for males and females, needs to be considered and that consideration of species-specific factors indicates multiple causes of pair-living among mammals.
